# Factors Affecting Arsenic Methylation in Arsenic-Exposed Humans: A Systematic Review and Meta-Analysis 

**DOI:** 10.3390/ijerph13020205

**Published:** 2016-02-06

**Authors:** Hui Shen, Qiang Niu, Mengchuan Xu, Dongsheng Rui, Shangzhi Xu, Gangling Feng, Yusong Ding, Shugang Li, Mingxia Jing

**Affiliations:** Department of Public Health, Shihezi University School of Medicine, Shihezi 832000, Xinjiang, China; 18240933220@163.com (H.S.); shzniuqiang@sina.com (Q.N.); 18097533626@163.com (M.X.); ruidongsheng@gmail.com (D.R.); shzxushangzhi@sohu.com (S.X.); 18139260682@189.cn (G.F.); tianmajuechen@alijun.com (Y.D.); jingmingxia211@163.com (M.J.)

**Keywords:** arsenic, methylation, meta-analysis, human

## Abstract

Chronic arsenic exposure is a critical public health issue in many countries. The metabolism of arsenic *in vivo* is complicated because it can be influenced by many factors. In the present meta-analysis, two researchers independently searched electronic databases, including the Cochrane Library, PubMed, Springer, Embase, and China National Knowledge Infrastructure, to analyze factors influencing arsenic methylation. The concentrations of the following arsenic metabolites increase (*p*< 0.000001) following arsenic exposure: inorganic arsenic (iAs), monomethyl arsenic (MMA), dimethyl arsenic (DMA), and total arsenic. Additionally, the percentages of iAs (standard mean difference (SMD): 1.00; 95% confidence interval (CI): 0.60–1.40; *p*< 0.00001) and MMA (SMD: 0.49; 95% CI: 0.21–0.77; *p* = 0.0006) also increase, while the percentage of DMA (SMD: −0.57; 95% CI: −0.80–−0.31; *p*< 0.0001), primary methylation index (SMD: −0.57; 95% CI: −0.94–−0.20; *p* = 0.002), and secondary methylation index (SMD: −0.27; 95% CI: −0.46–−0.90; *p* = 0.004) decrease. Smoking, drinking, and older age can reduce arsenic methylation, and arsenic methylation is more efficient in women than in men. The results of this analysis may provide information regarding the role of arsenic oxidative methylation in the arsenic poisoning process.

## 1. Introduction

Arsenic is a toxic metalloid element that is ubiquitous in the environment. The World Health Organization (WHO) and the International Agency for Research on Cancer (IARC) have identified it and its compounds as human carcinogens [1,2]. Arsenic enters an organism via the respiratory tract, alimentary canal, and skin, and it is primarily metabolized in the liver [3]. The metabolic pattern has primarily been regarded as occurring through oxidative methylation [4], which was formerly considered a detoxification pattern [5]. However, monomethylarsonous acid (MMA^III^) and dimethylarsonous acid (DMA^III^) have recently been shown to be more poisonous than inorganic arsenic (iAs) [6,7]. Therefore, arsenic toxicity is closely related to its metabolism, which in turn is highly dependent on the methylation status and valence states of its metabolites. Recently, research has increasingly focused on the factors influencing arsenic methylation. In addition to the dosage of arsenic exposure, an individual’s ethnicity [8], age, sex [9], body mass index (BMI) [10], lifestyle and dietary history [11], and inherited genetic characteristics [12] were also related with the arsenic methylation capacity. Nonetheless, the results of some studies contrast the studies described in this paragraph [13,14]. Thus, the factors reported to influence arsenic methylation differ among studies. We are unaware of a systematic review or meta-analysis for this topic. Therefore, we performed a systematic review and meta-analysis of the literature to comprehensively analyze the relevant data regarding the effect of arsenic exposure and factors related with arsenic methylation. This work provides a reference for the role of arsenic oxidative methylation in the arsenic poisoning process. 

## 2. Experimental Section 

A meta-analysis is appropriate for this purpose because it integrates the results from current evidence; it is particularly relevant for controversial research issues. 

### 2.1. Search Strategy

Searches were performed using the following electronic databases: the Cochrane Library, PubMed, Springer, Web of Science, China Science and Technology Journal Database (CSTJ), and China National Knowledge Infrastructure (CKNI) (last search updated in September 2015). The key search string was ((human OR person OR people) AND (arsenic OR As)) AND methylation.

### 2.2. Eligibility Criteria

To be eligible, the study had to be a cross-sectional study, case-control study, or cohort study in humans published in either Chinese or English. The factors investigated had to include arsenic exposure (drinking water, inhalation, or diet), sex, age, smoking status, drinking status, and BMI. The indicators of arsenic methylation capacity included concentration and percentage of arsenic metabolites, the primary methylation index (PMI), and the secondary methylation index (SMI).

### 2.3. Exclusion Criteria

The exclusion criteria were as follows: no report of indexes, such as concentrations or percentages of arsenic metabolites, PMI, or SMI; unavailable data (e.g., the data were depicted only in figures or were provided using means and/or ranges); duplicate publications; and reviews.

### 2.4. Outcome Indicators

Outcome indicators were the concentrations of inorganic arsenic (iAs), monomethyl arsenic (MMA), dimethyl arsenic (DMA), and total arsenic (TAs). The percentages of inorganic arsenic (iAs%), monomethyl arsenic (MMA%), and dimethyl arsenic (DMA%) were defined as iAs/TAs × 100%, MMA/TAs × 100%, and DMA/TAs × 100%, respectively. Two methylation indices, PMI ((MMA+DMA)/TAs or MMA/iAs) and SMI (DMA/(MMA+DMA) or DMA/MMA), were calculated to assess arsenic methylation capacity.

### 2.5. Data Extraction and Article Quality Evaluation

Two reviewers independently screened full-length articles. The following information was extracted from the full text of each qualified study: publication characteristics (title of the study, first author, publication date, and journal/magazine), baseline data (*n*, mean ± standard deviation (SD)) for the experimental and control groups, subject characteristics (sex, age, BMI, nationality, dosage and pattern of exposure, and smoking and drinking status), outcome indicators, and the source of indicator estimates. If the two reviewers disagreed, a third party (a university professor who is an expert and lecturer on meta-analyses) made the final decision.

The Australian Joanna Briggs Institute (JBI) standard, which is the accepted standard for assessing a study’s quality, was used in the present study for study quality assessment. There are 3 grades for each of the 10 items: 2, detailed description of the item; 1, only a mention of the item but no detail; and 0, nothing reported for the item.

### 2.6. Data Analysis

Twenty-five articles were analyzed in Review Manager Version 5.2 (The Nordic Cochrane Centre, The Cochrane Collaboration, 2012, Portland Oregon, OR, USA) and Stata 12.0 (StataCorp, College Station, Texas, TX, USA). To assess the heterogeneity among studies, we calculated the *I*^2^ [15]. Low, medium, and high levels of heterogeneity were considered as *I*^2^ < 40%, 40%–60%, and >60%, respectively. We use a fixed model on the conditions of *p* > 0.05 and *I*^2^ < 40%. Otherwise, a randomized model was used to estimate the combined effect. A multivariate meta-regression analysis was performed to determine the source of heterogeneity. Continuous variables were estimated as standardized mean differences with 95% confidence intervals (CIs). All reported *p*-values are two-sided, and a significance level of 0.05 was used. For additional insight, subgroup analyses were performed based on nationality (Asian or American), age (children or adults), sex constituent ratio (≤50% men or >50% men), and arsenic exposure dosage (low exposure (≤50 µg/L) or high exposure (>50 µg/L)). Small study effects were explored using funnel plots and Egger’s tests. Sensitivity analyses were performed using Stata 12.0.

## 3. Results

### 3.1. Study Characteristics

**Figure 1:**
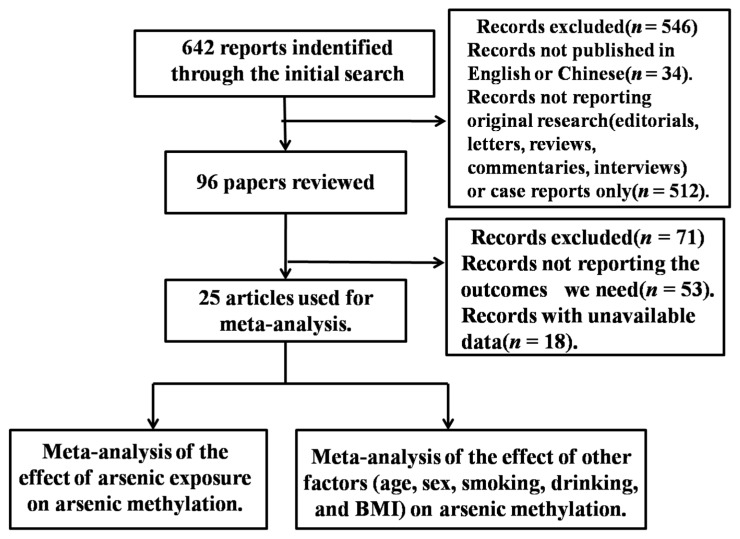
Flowchart of search strategy. BMI: body mass index

Using the search strategy, 642 articles were identified (Figure 1), of which 25 were used for the meta-analysis based on the eligibility and exclusion criteria [8,16,17,18,19,20,21,22,23,24,25,26,27,28,29,30,31,32,33,34,35,36,37,38,39]. Humans were the subjects, and the exposure modes included daily exposure, such as through drinking water or diet, or occupational exposure. Urine samples were collected for analysis from the participants in these studies. 

Based on the JBI criteria, all of the included papers were of high quality, as demonstrated by scores >12 (Table 1).

**Table 1:** Quality of the included studies, asevaluated using the Joanna Briggs Institute criteria.

Author Year	The Purpose of Research is Clear, and the Basis is Sufficient	Subjects were Identified Using Random Sampling	Defined the Inclusion Criteria Clearly	Took Suitable Measures to Avoid Confounders	Evaluated the Results Using an Objective Standard	Took Suitable Measures to Check the Authenticity of the Information	Considered the Ethical Issues in the Studies	Used the Right Statistical Methods	The Statement and Analysis of Results are Appropriate	The Research Value was Expounded Clearly	Total
Ming Zhang 2011 [29]	1	2	1	2	2	0	1	1	2	1	13
Haixu Wang 2009 [28]	2	0	1	2	2	1	2	1	1	2	14
Yu-Mei Hsueh 2003 [19]	2	1	2	2	2	2	1	1	2	2	17
Yuanyuan Xu 2008 [25]	2	0	2	1	2	2	2	1	2	2	16
Yuanyuan Xu 2009 [27]	2	0	2	1	2	1	2	1	2	2	15
Xin Li 2013 [31]	2	1	2	1	2	2	2	2	2	1	17
Xin Li 2008 [24]	2	1	2	2	2	1	1	1	2	1	15
Pantip Hinhumpatch 2013 [30]	2	0	1	1	2	1	2	2	2	2	15
L. M. Del Razo 1997 [18]	2	1	2	2	2	1	2	2	2	2	18
Julia E Heck 2007 [20]	2	1	2	2	2	1	2	2	2	2	18
Hung-Yi Chiou 1997 [17]	2	0	1	1	2	1	1	1	2	1	12
Habibul Ahsan 2007 [21]	2	2	1	1	2	2	2	2	2	2	18
Guifan Sun 2007 [22]	2	1	1	2	2	2	2	1	2	2	17
Claudia Hopenhayn-Rich 1996 [16]	2	1	1	2	2	2	2	2	2	2	18
Christopher A. Loffredo 2003 [8]	2	0	1	1	2	1	1	2	2	2	14
Chi-Jung Chung 2009 [26]	2	1	2	1	2	2	1	2	2	2	17
Beata Janasik 2015 [32]	2	1	2	2	2	1	2	1	2	1	16
Alba Herna´ndez 2008 [23]	2	2	1	2	2	2	2	1	2	2	18
Chin-Hsiao Tseng 2005 [33]	2	1	1	2	2	2	1	1	2	2	16
Craig Steinmaus 2010 [36]	2	1	1	2	2	1	2	2	2	2	17
Dawit Melak 2013 [37]	2	2	1	2	2	1	2	2	2	2	18
Qiang Zhang 2013 [38]	2	1	2	1	2	2	2	2	2	2	18
Ya-Li Huang 2009 [35]	2	1	2	1	2	2	1	1	2	2	16
Yung-Kai Huang 2007 [34]	2	1	2	1	2	2	1	1	2	2	16
Yung-Kai Huang 2008 [39]	2	1	1	1	2	2	1	2	2	2	16

### 3.2. Meta-Analysis of Arsenic Exposure Effects

#### 3.2.1. Effect of Arsenic Exposure on Total Arsenic

**Figure 2:**
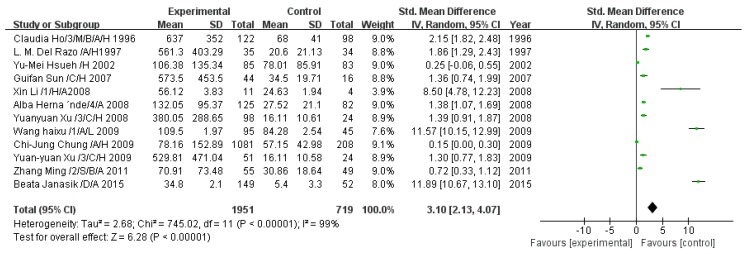
Effect of arsenic exposure on total arsenic (TAs) concentration in urine. Std., standardized; SD, standard deviation; IV, independent variable; CI, confidence interval.

A total of 12 studies estimated TAs concentration. The pooled analysis showed that the TAs concentration was 3.10-fold higher in the exposed group than in the control group (95% CI, 2.13–4.07; *Z* = 6.28; *p* < 0.00001), with significant heterogeneity (*p* < 0.00001; *I*^2^ = 99%; Figure 2).

#### 3.2.2. Effect of Arsenic Exposure on Inorganic Arsenic

**Figure 3:**
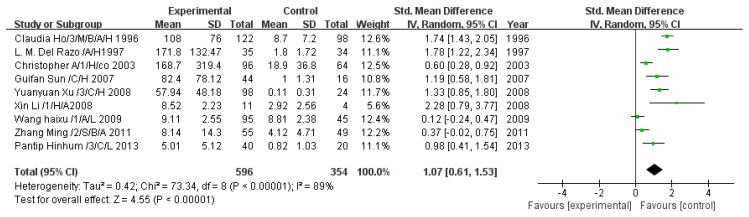
Effect of arsenic exposure on inorganic arsenic (iAs) concentration in urine. Std., standardized; SD, standard deviation; IV, independent variable; CI, confidence interval.

A total of nine studies estimated iAs concentration. The pooled analysis showed that iAs in the exposed group was 1.07-fold higher than that in the control group (95% CI, 0.61–1.53; *Z* = 4.55; *p* < 0.00001), with significant heterogeneity (*p* < 0.00001; *I*^2^ = 89%; Figure 3).

#### 3.2.3. Effect of Arsenic Exposure on Monomethyl Arsenic

**Figure 4:**
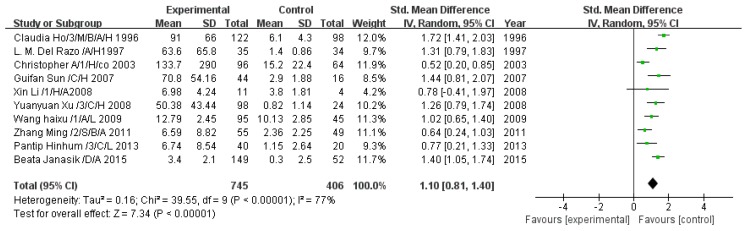
Effect of arsenic exposure on monomethyl arsenic (MMA) concentration in urine. Std., standardized; SD, standard deviation; IV, independent variable; CI, confidence interval.

A total of 10 studies estimated MMA concentration. The pooled analysis showed that the MMA concentration in the exposed group was 1.10-fold higher than that in the control group (95% CI, 0.81–1.40; *Z* = 7.34; *p* < 0.00001), with significant heterogeneity (*p* < 0.00001; *I*^2^ = 77%; Figure 4).

#### 3.2.4. Effect of Arsenic Exposure on Dimethyl Arsenic

**Figure 5:**
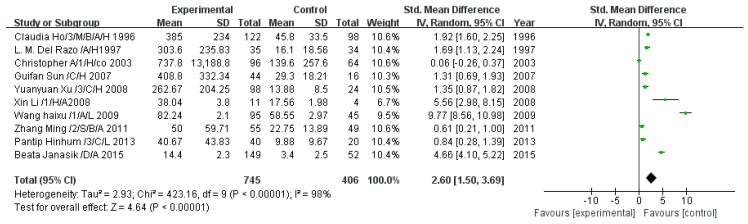
Effect of arsenic exposure on dimethyl arsenic (DMA) concentration in urine. Std., standardized; SD, standard deviation; IV, independent variable; CI, confidence interval.

A total of 10 studies estimated DMA concentration. The pooled analysis showed that the DMA concentration in the exposed group was 2.60-fold higher than that in the control group (95% CI, 1.50–3.69; *Z* = 4.64; *p* < 0.00001), with significant heterogeneity (*p* < 0.00001; *I*^2^ = 98%; Figure 5).

#### 3.2.5. Effect of Arsenic Exposure on Inorganic Arsenic Percentage

**Figure 6:**
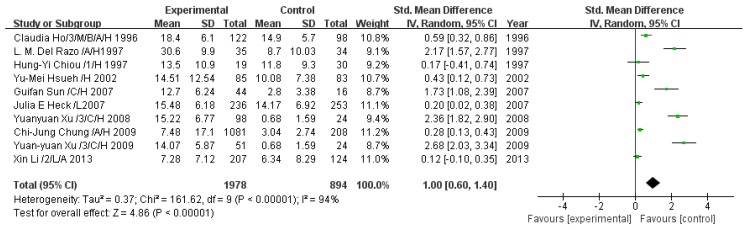
Effect of arsenic exposure on inorganic arsenic percentage (iAs%) in urine. Std., standardized; SD, standard deviation; IV, independent variable; CI, confidence interval

A total of 10 studies estimated iAs%. The pooled analysis showed that iAs% in the exposed group was 1.00-fold higher than that in the control group (95% CI, 0.60–1.40; *Z* = 4.86; *p* < 0.00001), with significant heterogeneity (*p* < 0.00001; *I*^2^ = 94%; Figure 6).

#### 3.2.6. Effect of Arsenic Exposure on Monomethyl Arsenic Percentage

**Figure 7:**
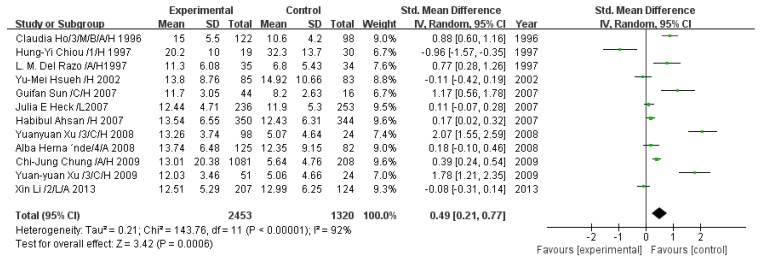
Effect of arsenic exposure on monomethyl arsenic percentage (MMA%) in urine. Std., standardized; SD, standard deviation; IV, independent variable; CI, confidence interval

A total of 12 studies estimated MMA%. The pooled analysis showed that MMA% in the exposed group was 0.49-fold higher than that in the control group (95% CI, 0.21–0.77; *Z* = 3.42; *p* = 0.0006), with significant heterogeneity (*p* < 0.00001; *I*^2^ = 92%; Figure 7).

#### 3.2.7. Effect of Arsenic Exposure on Dimethyl Arsenic Percentage

**Figure 8:**
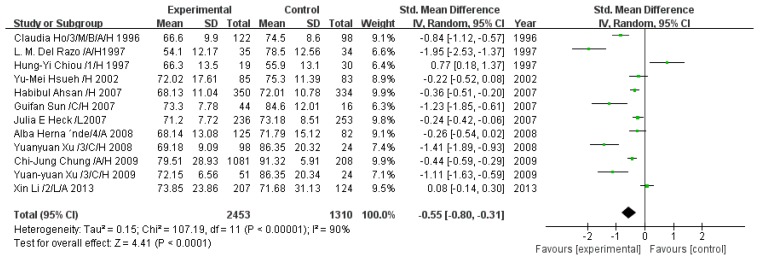
Effect of arsenic exposure on dimethyl arsenic percentage (DMA%) in urine. Std, standardized; SD, standard deviation; IV, independent variable; CI, confidence interval

A total of 12 studies estimated DMA%. The pooled analysis showed that DMA% in the exposed group was 0.55-fold lower than that in the control group (95% CI, 0.80–0.31; *Z* = 4.41; *p* < 0.0001), with significant heterogeneity (*p* < 0.00001; *I*^2^ = 90%; Figure 8).

#### 3.2.8. Effect of Arsenic Exposure on the Primary Methylation Index

**Figure 9:**
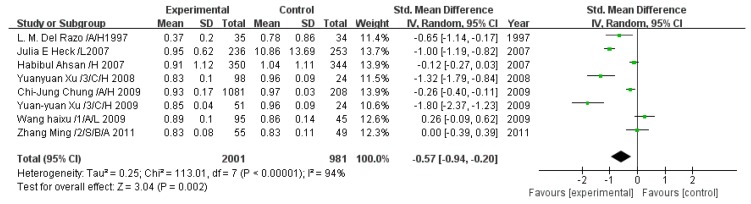
Effect of arsenic exposure on the primary methylation index (PMI). Std., standardized; SD, standard deviation; IV, independent variable; CI, confidence interval.

A total of nine studies estimated the PMI. The pooled analysis showed that the PMI in the exposed group was 0.57-fold lower than that in the control group (95% CI, 0.94–0.20; *Z* = 3.04; *p* = 0.002), with significant heterogeneity (*p* < 0.00001; *I*^2^ = 94%; Figure 9).

#### 3.2.9. Effect of Arsenic Exposure on the Secondary Methylation Index

**Figure 10:**
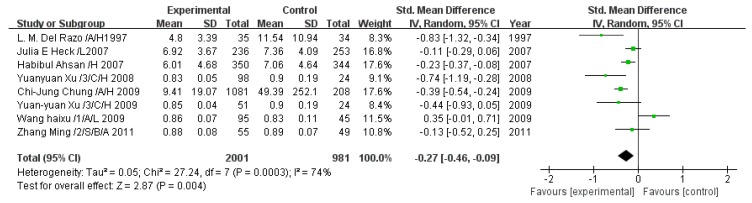
Effect of arsenic exposure on the secondary methylation index (SMI). Std., standardized; SD, standard deviation; IV, independent variable; CI, confidence interval

A total of nine studies estimated the SMI. The pooled analysis showed that the SMI in the exposed group was 0.27-fold lower than that in the control group (95% CI, 0.46–0.09; *Z* = 2.87; *p* = 0.004), with significant heterogeneity (*p* = 0.0003; *I*^2^ = 74%; Figure 10).

### 3.3. Subgroup Analyses of the Effects of Arsenic Exposure

**Figure 11:**
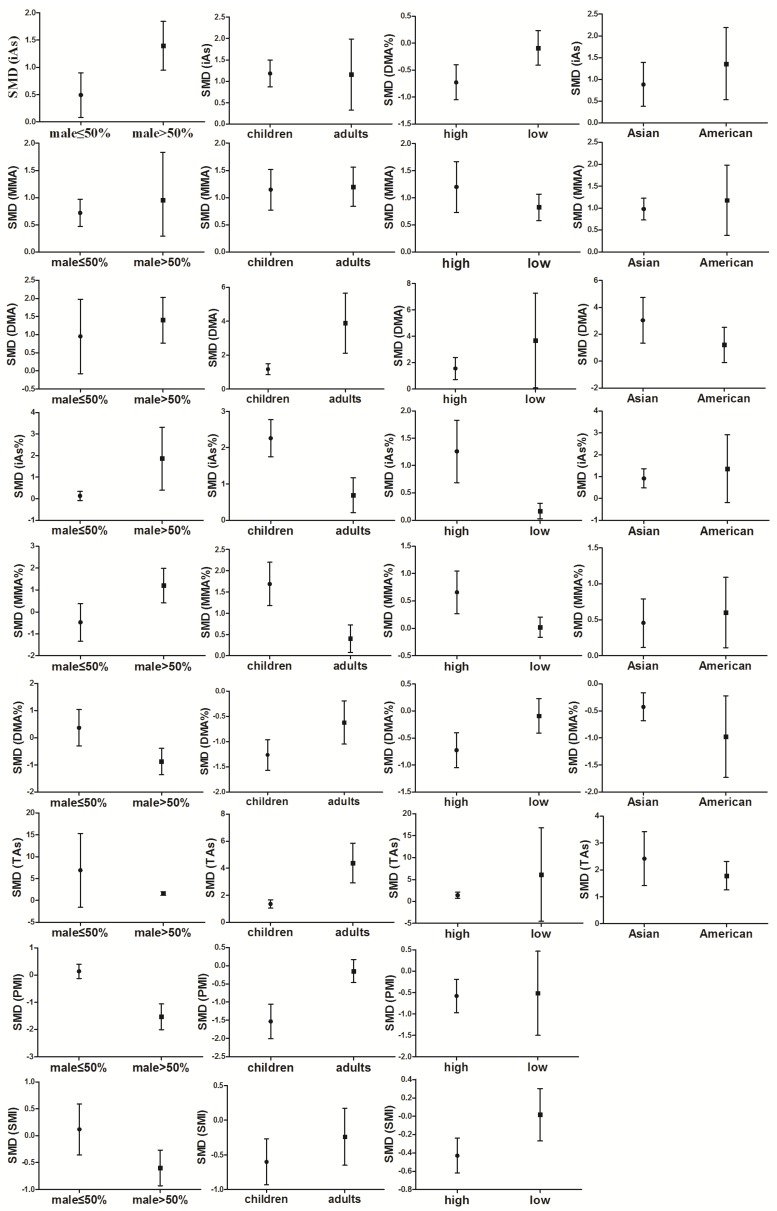
Results of the subgroup analyses of the effects of arsenic exposure (high *vs.* low). SMD, standardized mean difference; iAs, inorganic arsenic; MMA, monomethyl arsenic; DMA, dimethyl arsenic; iAs%, percentage of inorganic arsenic; MMA%, percentage of monomethyl arsenic; DMA%, percentage of dimethyl arsenic; TAs, total arsenic; PMI, primary methylation index; SMI, secondary methylation index.

In the subgroup analyses, the differences in iAs% and MMA% between the high and low exposure groups were higher in children than in adults (*p* < 0.00001; Figure 11), while the differences in TAs, DMA, and PMI between the high and low exposure groups were lower in children than in adults (*p* < 0.0001). Based on sex ratio, groups with >50% men had greater iAs (*p* = 0.003), iAs% (*p* = 0.002), and MMA% (*p* = 0.02) but smaller DMA% (*p* = 0.01) and PMI (*p* < 0.00001) than the groups that were ≤50% male. The high-exposure groups higher iAs, iAs%, and MMA% than the low-exposure groups. Furthermore, changes in iAs, MMA, DMA, iAs%, and DMA% were observed in the subjects exposed to drinking water with <50 µg/L arsenic. Significant differences between Asians and Americans were not detected.

### 3.4. Meta-Analysis of the Effects of Other Factors 

#### 3.4.1. Effect of Sex on Arsenic Methylation

**Figure 12:**
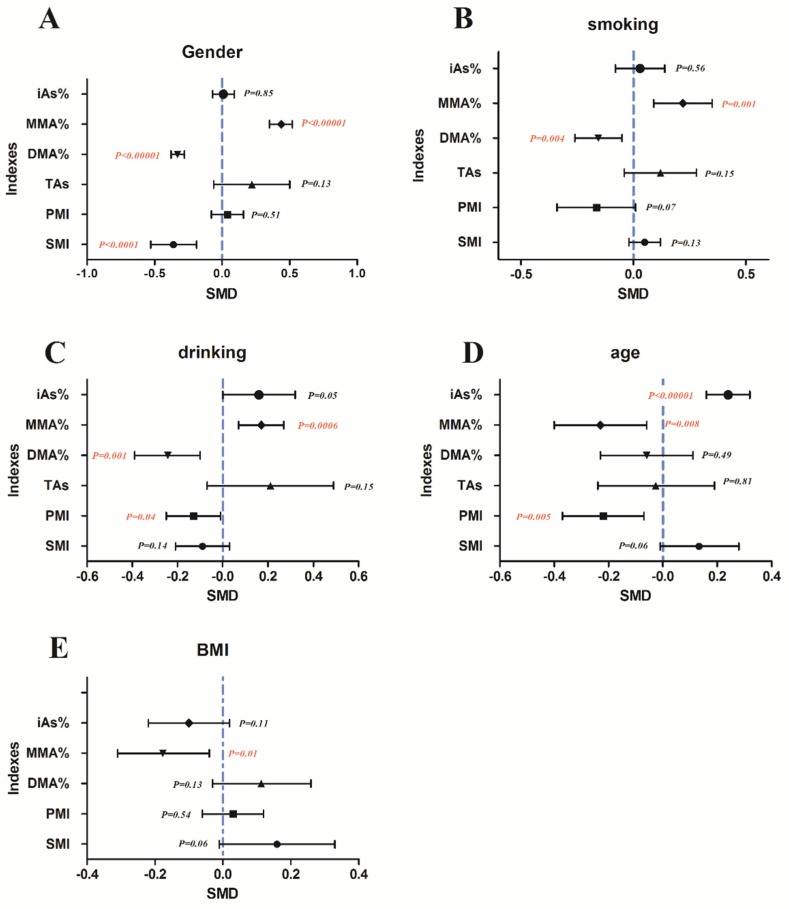
Effect of non-exposure factors on arsenic methylation (difference between (**A**) sexes, (**B**) smoking; (**C**) alcohol; (**D**) age; and (**E**) body mass index (BMI) on the arsenic methylation indexes, including the percentage of inorganic arsenic (iAs%), percentage of monomethyl arsenic (MMA%), percentage of dimethyl arsenic (DMA%), total arsenic (TAs), primary methylation index (PMI), and secondary methylation index (SMI). SMD, standardized mean difference

A total of 12 studies compared the MMA% between men and women. The pooled analysis showed that the MMA% in men was 0.44-fold higher than in women (95% CI, 0.35–0.52; *Z* = 10.37; *p* < 0.00001), with significant heterogeneity (*p* < 0.00001; *I*^2^ = 52%; Figure 12A). A total of 12 studies compared the DMA% between men and women. The pooled analysis showed that the DMA% was 0.33-fold lower in men than in women (95% CI, 0.38–0.28; *Z* = 12.35; *p* < 0.00001), with significant heterogeneity (*p* = 1; *I*^2^ = 0%). A total of six studies compared the SMI between men and women. The pooled analysis showed that the SMI was 0.36-fold lower in men than in women (95% CI, 0.53–0.19; *Z* = 4.10; *p* < 0.0001), with significant heterogeneity (*p* < 0.00001; *I*^2^ = 86%).

#### 3.4.2. Effect of Smoking on Arsenic Methylation

A total of 11 studies compared the MMA% by smoking status. The pooled analysis showed that the MMA% was 0.22-fold higher in smokers than in non-smokers (95% CI, 0.09–0.35; *Z* = 3.25; *p* = 0.001), with significant heterogeneity (*p* < 0.00001; *I*^2^ = 78%; Figure 12B). A total of 11 studies compared the DMA% by smoking status. The pooled analysis showed that the DMA% was 0.16-fold lower among smokers than among non-smokers (95% CI, 0.26–0.05; *Z* = 2.89; *p* = 0.004), with significant heterogeneity (*p* = 0.001; *I*^2^ = 65%).

#### 3.4.3. Effect of Drinking Alcohol on Arsenic Methylation

A total of seven studies compared the iAs% by alcohol consumption. The pooled analysis showed that the iAs% was 0.16-fold higher among drinkers than among non-drinkers (95% CI, 0.00–0.32; Z = 1.97; *p* = 0.05), with significant heterogeneity (*p* = 0.02; *I*^2^ = 61%; Figure 12C). A total of seven studies compared the MMA% by alcohol consumption. The pooled analysis showed that the iAs% among drinkers was 0.17-fold higher than that among non-drinkers (95% CI, 0.07–0.27; *Z* = 3.45; *p* = 0.0006), with significant heterogeneity (*p* = 0.78; *I*^2^ = 0%). A total of 7 studies compared the DMA% by alcohol consumption. The pooled analysis showed that the DMA% was 0.24-fold lower among drinkers than among non-drinkers (95% CI, 0.39–0.10; *Z* = 3.26; *p* = 0.001), with significant heterogeneity (*p* = 0.05; *I*^2^ = 53%). A total of four studies compared the PMI by alcohol consumption. The pooled analysis showed that the PMI in drinkers was 0.13-fold lower than that in non-drinkers (95% CI, 0.25–0.01; *Z* = 2.06; *p* = 0.04), with significant heterogeneity (*p* = 0.22; *I*^2^ = 31%).

#### 3.4.4. Effect of Age on Arsenic Methylation

A total of seven studies compared the iAs% by age. The pooled analysis showed that the iAs% among subjects aged ≤50 years was 0.24-fold higher than that among subjects aged >50 years (95% CI, 0.16–0.32; *Z* = 5.88; *p* < 0.00001), with significant heterogeneity (*p* = 0.14; *I*^2^ = 38%; Figure 12D). A total of eight studies compared the MMA% by age. The pooled analysis showed that the MMA% among subjects aged ≤50 years was 0.23-fold lower than that among subjects aged >50 years (95% CI, 0.40–0.06; *Z* = 2.66; *p* = 0.008), with significant heterogeneity (*p* < 0.0001; *I*^2^ = 80%); A total of five studies compared the PMI by age. The pooled analysis showed that the PMI for subjects aged ≤50 years was 0.22-fold lower than that for participants aged >50 years (95% CI, 0.37–0.07; *Z* = 2.83; *p* = 0.005), with significant heterogeneity (*p* = 0.01; *I*^2^ = 69%).

#### 3.4.5. Effect of Body Mass Index on Arsenic Methylation

A total of four studies estimated the MMA% based on BMI. The pooled analysis showed that the MMA% with a high BMI was 0.18-fold lower than that with a low BMI (95% CI, 0.31–0.04; *Z* = 2.55; *p* = 0.01), with significant heterogeneity (*p* = 0.01; *I*^2^ = 52%; Figure 12E).

### 3.5. Small-Study Effect Evaluation 

**Figure 13:**
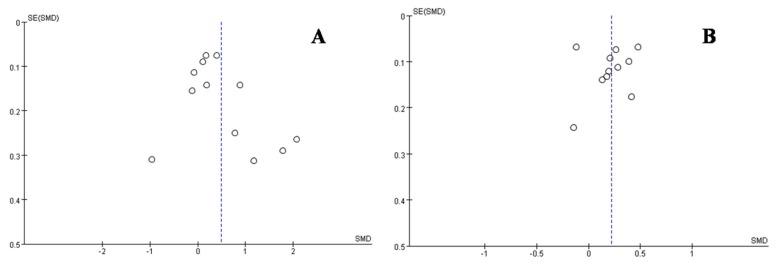
Funnel plot for the percentage of monomethyl arsenic (MMA%) that was affected by both arsenic exposure (**A**) and smoking (**B**). Blue-dotted line shows the overall estimated standard mean difference. Evidence for publication bias was not found. SMD, standard mean difference; SE, standard error.

Visual inspection of the funnel plot and Egger’s test results showed no evidence of significant small-study effects (*p_A_* = 0.550, *p_B_* = 0.921) (Figure 13).

### 3.6. Sensitivity Analysis 

**Figure 14:**
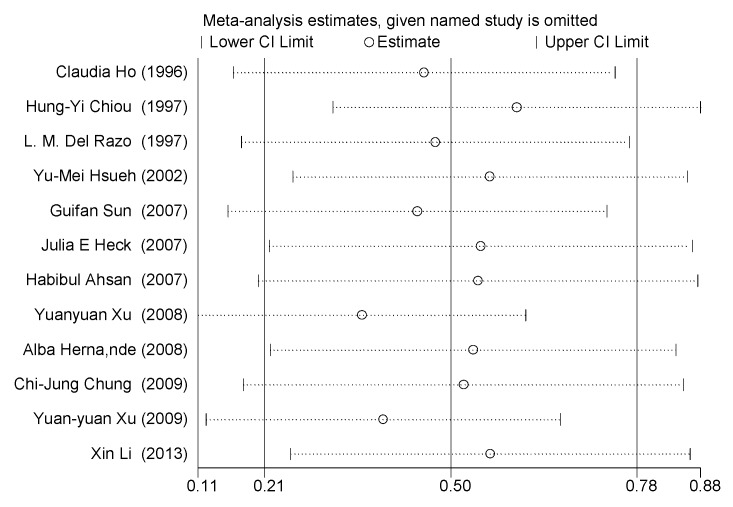
Sensitivity analysis for the percentage of monomethyl arsenic, which was affected by arsenic exposure. CI, confidence interval.

We conducted a sensitivity analysis for the MMA%. As shown in Figure 14, all of the included studies were distributed evenly from the central line, and none of the studies deviated significantly. Therefore, no individual study influenced the combined results.

### 3.7. Meta-Regression Analysis of Arsenic Exposure Effects

A meta-regression analysis was performed with the MMA% as the Y (outcome) and age (children *vs.* adults), sex constituent ratio (>50% men *vs.* ≤50% men), nationality (Asian *vs.* American), and exposure dose (low exposure (≤50 µg/L) *vs.* high exposure (>50 μg/L)) as the X variables (factors). Age, as included in this regression model, provided more evidence to illustrate the relationship between age and arsenic methylation (Table 2).

**Table 2:** Results of the meta-regression analysis for the percentage of monomethyl arsenic

Item	Coefficient	Standard Error	t	*p*	95% CI	
Age	−0.90	0.17	−5.35	0.00	−1.30	−0.50
Sex	0.37	0.17	2.11	0.07	−0.04	0.78
Dose	0.01	0.28	0.05	0.96	−0.65	0.68
Country	−0.31	0.34	−0.91	0.39	−1.12	0.50

## 4. Discussion

The present results, based on a meta-analysis of the current best evidence, indicate that the dose of arsenic exposure is negatively related with arsenic methylation capacity and women have a better methylation capacity than men. Smoking and drinking, and potentially age and BMI, are also associated with poorer methylation capacity. 

**Figure 15:**
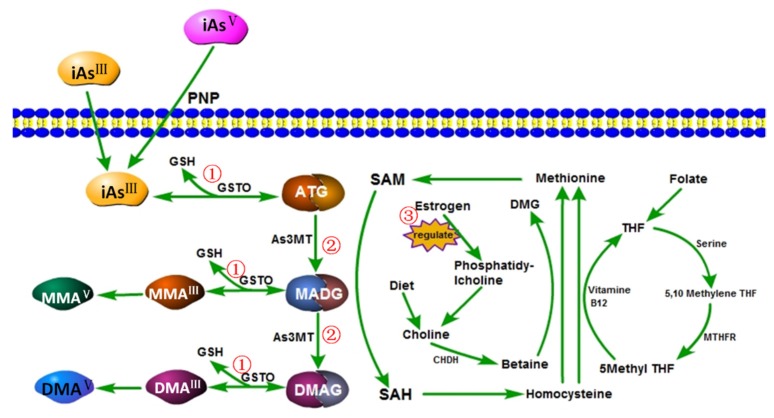
Metabolic pathways of arsenic methylation and one-carbon metabolism. iAs^III^, inorganic; iAs^V^, inorganic arsenate; MMA^III^, monomethylarsonous acid; MMA^V^, Monomethylarsonic acid; DMA^III^, dimethylarsinous; DMA^V^, dimethylarsinic acid; ATG, arsenic triglutathione; MADG, monomethylarsenic diglutathione; DMAG, dimethylarsinic glutathione; PNP, purine nucleoside phosphorylase; GSH, reduced glutathione; GSTO, glutathione S-transferase omega; SAM, S-adenosylmethionine; SAH, S-adenosylhomocysteine; CHDH, choline dehydrogenase; DMG, dimethylglycine; THF, tetrahydrofolate; MTHF, methylenetetrahydrofolate. 1 and 2: arsenic exposure might lead to insufficient metabolic-associated factors or enzymes, such as sadenosylmethionine (SMA) and glutathione (GSH); 3: estrogen can indirectly promote arsenic metabolism by up-regulating betaine formed by choline oxidation.

Recent studies suggest that exposure to arsenic may increase the risk of certain diseases, including arsenic-induced skin lesions [40], peripheral artery disease [41], hypertension [42,43], cardiovascular disease [44], diabetes [45], skin cancer and urothelial cancer [46], and reproductive system damage [47]. The primary mode of arsenic metabolism is generally considered to be methylation (Figure 15). However, the toxicities and targets of iAs^III^, iAs^V^, MMA^III^, MMA^V^, DMA^III^, and DMA^V^ that are produced in the metabolic process are not consistent. 

Although the average urinary values for iAs%, MMA%, and DMA% among arsenic-exposed people are 10%–30%, 10%–20%, and 60%–70% respectively, there are differences between individuals. Epidemiological studies suggest that high MMA%, low DMA%, and low SMI are associated with the incidence of arsenic-related diseases [33,34,48,49]. In the present study, arsenic exposure led to high levels of iAs% and MMA% and low levels of DMA%, FMR, and SMR, indicating inefficient methylation. The changes in the subgroup exposed to drinking water with arsenic >100 μg/L were more significant. Those results suggest that the arsenic methylation capacity declines with increased doses of exposure, implying that highly toxic iAs^III^ and MMA^III^ at the cellular or blood level may lead to arsenic-related injuries. Continued arsenic exposure may cause insufficient metabolic-associated factors or enzymes such as S-adenosylmethionine and glutathione (Figure 15, 1 and 2). Additionally, a considerable amount of glutathione is consumed by oxidative stress. It is worth noting that inefficient methylation has also been found in a low-exposure subgroup (<50 μg/L); that is, chronic low-exposure might also be deleterious for health. 

The subgroup analysis showed that children had a lower methylation capacity than adults, which may be due to metabolism-related organs and enzymes during growth in children. In contrast, Chowdury *et al.* [50] suggested that children might have better methylation capacity, but the study lacked sufficient statistical data. Therefore, further studies are required to confirm the differences between children and adults. 

Although no significant differences were detected between Asians and Americans, remarkable differences in the distribution of urinary arsenic species were detected among Chinese, Chileans, and Mexicans; however, the sample sizes of Chinese and Chilean participants were too small [8]. Another study reported ethnic differences in arsenic methylation capacity [16]; indigenous people in Chile had better methylation than individuals of European ethnic origin. Smith *et al.* [51] suggested that indigenous peoples have less severe clinical manifestations and the ethnic differences were derived from the different genetic locus of arsenic-metabolizing enzymes.

Women had better methylation capacities than men in not only the subgroup analyses of arsenic exposure but also the meta-analysis of sex effects. One possible mechanism is that betaine formed from choline oxidation can donate its methyl group to homocysteine to form methionine. Then, choline can be derived from phosphatidylcholine, which is up-regulated by estrogen [52]. Thus, estrogen may indirectly promote arsenic metabolism (Figure 15, 3). Better methylation capacity and the effect of estrogen in were present for women of childbearing age and not adolescents [53]. In addition, men might be exposed to more factors that inhibit arsenic methylation, such as drinking and smoking.

Smoking was associated with low methylation capacity in the present study, supporting a previous study, in which inefficient methylation and cardiovascular disease were related with older age and smoking [54]. Both smoking and arsenic exposure can stimulate cells to release free radicals and consume antioxidants, resulting in oxidative stress and tissue damage [55]. In addition, some chemicals in cigarettes can influence the enzymes involved in the methylation processes, especially those involved in the second methylation phase. Moreover, smoking itself could be a pathway for arsenic exposure if the cigarettes contain trace amounts of arsenic. Alcohol consumption could affect the methylation processes, because alcohol results in liver damage, which is the primary organ associated with arsenic metabolism. Because smoking and drinking are more common behaviors in men than in women, sex, smoking status, and drinking status might confound each other.

In the meta-analysis of the effect of age on arsenic methylation, MMA% and PMI increased with age; SMI was somewhat, but not significantly, higher. Similarly, Huang *et al.* reported that MMA% increased, while both DMA% and SMI decreased [39]. Higher MMA% and lower DMA% and SMI are potentially risk factors for arsenicosis. However, there is little evidence that either iAs% or PMI is related with arsenicosis [56]. Therefore, older people might have poorer methylation capacity and are susceptible to arsenic damage. Many organs are senescent, which may block the methylation process, and older age might also be associated with longer arsenic exposure. The present study also indicated that higher BMIs were associated with a lower MMA%, suggesting that BMI might be positively related with methylation ability. Further studies regarding the relationship between the key molecular factors and arsenic methylation are needed.

## 5. Conclusions

In summary, the methylation of arsenic is influenced by a variety of factors. Arsenic exposure, smoking, drinking, and older age can reduce the capacity of arsenic methylation. Furthermore, arsenic methylation is more efficient in women than in men. The present study was limited by the obvious heterogeneity in the data. Although the heterogeneity was diminished in the subgroup analyses, it remained high. This heterogeneity might have been related with the variation in a number of characteristics among the studies, such as ethnicity, exposure duration, nutrition and dietary factors, and lifestyle. However, there was no evidence of significant publication bias, and the sensitivity of the articles was satisfactory. Thus, the method was suitable to analyze the relationship between the factors and arsenic methylation, resulting in reliable conclusions.
